# A population based perspective on children and youth with brain tumours

**DOI:** 10.1186/s12885-015-2016-0

**Published:** 2015-12-23

**Authors:** Vincy Chan, Jason D. Pole, Robert E. Mann, Angela Colantonio

**Affiliations:** 1 Toronto Rehabilitation Institute, University Health Network, 550 University Avenue, Toronto, ON M5G 2A2 Canada; 2 Rehabilitation Sciences Institute, University of Toronto, 500 University Avenue, Toronto, ON M5G 1V7 Canada; 3 Pediatric Oncology Group of Ontario, 480 University Avenue, Toronto, ON M5G 1V2 Canada; 4 Centre for Addiction and Mental Health, 33 Russell Street, Toronto, ON M5S 2S1 Canada

**Keywords:** Brain tumours, International classification of diseases, Surveillance

## Abstract

**Background:**

There is currently no active surveillance of metastatic and non-malignant brain tumours in Canada as well as data on the health service use of children and youth with brain tumours. The objective of this study was to identify pediatric primary, metastatic, benign, and unspecified brain tumours in Ontario, Canada and to describe their health service use from a population based perspective.

**Methods:**

The population based healthcare administrative databases National Ambulatory Care Reporting System and the Discharge Abstract Database were used. Patients with malignant (primary and metastatic), benign, and unspecified brain tumours in acute care between fiscal year 2003/04 and 2009/10 were identified using specified International Classification of Diseases version ten codes.

**Results:**

Between fiscal year 2003/04 and 2009/10, there were 4022 brain tumour episodes of care (18.4 per 100,000 children and youth). Malignant brain tumors had the highest rates of episodes of care (14.9 times higher than that of benign and 5.7 times higher than that of unspecified brain tumours). Compared to patients with malignant brain tumours, those with benign brain tumours spent a longer period of time in acute care (*p* < .05) and patients with unspecified brain tumours stayed in the intensive care units for a longer period of time (*p* < .0001) with a lower proportion were discharged home (*p* < .0001).

**Conclusion:**

Despite higher rates of malignant brain tumour episodes of care, patients with benign and unspecified brain tumours also use acute care services and post-acute services that are currently not taken into account in healthcare planning and resource allocation. Active surveillance and research of metastatic and non-malignant brain tumours that can inform the planning of healthcare services and resource allocation for this population is encouraged.

**Electronic supplementary material:**

The online version of this article (doi:10.1186/s12885-015-2016-0) contains supplementary material, which is available to authorized users.

## Background

### A population based perspective on children and youth with brain tumours

Brain tumours can be malignant (primary and metastatic) or non-malignant. Primary brain tumours originate from within the brain and have the ability to spread within and invade the brain. Metastatic brain tumours originate from organs or tissue outside of the brain and, although less common among children and youth [1], they are the most common type of brain tumours overall (20–40 % of brain tumours) [2]. Non-malignant brain tumours (i.e., benign) do not contain cancer cells, are generally slow-growing, have well-defined borders, and do not invade surrounding tissue while malignant brain tumours are cancerous, fast growing, and can invade surrounding tissue and structures [3]. In Canada between 2000 and 2001, brain tumours accounted for $98.4 million in direct costs and $805.1 million in indirect costs and compared to all patients in Canada, those with primary brain tumours had longer median length of stay in acute care and higher readmission rates at one week and 1 month post-discharge [4].

To date, there is no such data on the number and health service use of pediatric patients with brain tumours in Ontario, Canada, even though primary brain tumours are one of the leading causes of cancer death in children and youth aged 19 years and under in Canada. The Canadian Cancer Society in Canada currently publishes yearly statistics on primary brain cancer [5]. However, there is a lack of detailed information such as temporal trends, geographic, and age specific data, as primary brain cancer is not one of the more common cancers overall (e.g., [5]). Further, it does not include information on metastatic or non-malignant brain tumours. In the province of Ontario, Canada, the Pediatric Oncology Group of Ontario provides data on pediatric brain cancer and captures all tumours regardless of behaviour (e.g., benign, uncertain, in situ, malignant) [6]. However, only patients that visit pediatric centres are captured. As a result, they do not capture all pediatric patients such as older adolescents that may be in an adult cancer facility. We acknowledge that the Brain Tumour Foundation of Canada has begun an initiative to establish a Canadian Brain Tumour Registry that counts every individual with a malignant or non-malignant brain tumour in Canada. As of early 2015, an implementation plan for this Registry was developed and as of May 2015, a plan was proposed to collect data from the provinces of Ontario, Manitoba, Alberta, British Columbia, and Quebec [7]. However, until such plan is put into action, there is no active surveillance of brain tumours that includes metastatic and non-malignant brain tumours in Canada and as such, no detailed information on primary brain tumours and equally important, no data on metastatic and non-malignant brain tumours in Ontario, Canada.

As survival rates continue to increase, data and surveillance of all types of brain tumours among children and youth is encouraged. Worldwide data on brain tumours have demonstrated the importance of including non-malignant brain tumours, including those that are uncertain or unspecified at admission, to reach a more accurate epidemiological profile of children and youth with brain tumours. For example, in Connecticut and Utah, United States, between 1985 and 1994, it was found that 12.1 and 10.3 % of all pediatric brain tumours were classified as benign and uncertain, respectively [8]. Across all age groups, the Tuscan Cancer Registry found that benign brain tumour age-adjusted annual incidence rate increased from 3.1 per 100,000 in 1985 to 6.1 per 100,000 in 2005 [9]. Finally, crude benign brain tumour incidence rates estimated from published data were found to range from 0.5 to 7.3 per 100,000 person-years [10]. However, an estimated rate for Canada was not available, as there are no published data available for the authors to include.

This paper is the first population based paper, to the best of our knowledge, to identify the healthcare utilization of all children and youth aged 19 years and under, hospitalized with malignant (primary and metastatic) and non-malignant brain tumours in Ontario, Canada between fiscal years 2003/04 and 2009/10. Specifically, the objectives of this paper were to identify the number, rates, and trends of children and youth with brain tumours and explore the patients’ demographic and clinical characteristics and their discharge destinations from acute care. It is important to recognize that non-malignant brain tumours can still result in long-term and serious consequences [11, 12], especially for a developing child. It has been suggested that, “given the absence of a cure [for metastatic brain tumors], rehabilitation and psychosocial services are crucial for both the patients and their families” [4]. As such, this paper has particular importance for the Canadian healthcare system, as it provides data on non-malignant brain tumours that have yet to be examined and identified. Findings can also help direct attention and resources to understand their impact on the healthcare system.

## Methods

### Data source

The Canadian Institute for Health Information (CIHI) National Ambulatory Care Reporting System (NACRS) and the Discharge Abstract Database (DAD) were used. The NACRS is a mandated data collection system that collects emergency department (ED) and ambulatory care data. Up to ten reasons for each visit to an ED in Ontario are included in the database [13]. The DAD contains all acute care hospital admissions and includes demographic and clinical information on all hospital admissions and discharges, including transfers and deaths, using standard diagnosis and procedure/intervention codes, in Ontario. A reabstraction study of the DAD indicated good agreement for non-clinical variables and moderate to substantial agreement for the most responsible diagnosis [14, 15]. Residents of Ontario, Canada have universal access to hospital-based care including ED and other ambulatory visits and as such, this study captured all patients with a brain tumour as identified using the case definition below between fiscal years 2003/04 and 2009/10 in Ontario, Canada.

### Case definition

Patients with brain tumours were identified in the NACRS and the DAD by the presence of specified International Classification of Diseases Version 10 codes (ICD-10) codes. The ICD, according to the World Health Organization, is ‘the standard diagnostic tool for epidemiology, health management, and clinical purposes’ and are used in healthcare administrative data to identify cases of interest [16]. Brain tumours were identified by and categorized based on the following ICD-10 codes: malignant (C70, C71, C79.3, C79.4), benign (D32.0, D33.0, D33.1, D33.2, D33.3), and unspecified (D42.0, D43.0, D43.1, D43.2).

### Variables

Demographic variables included age and sex. Children and youth aged 19 years and under were categorized into four age groups: 0–4 years (infants), 5–9 years (children), 10–14 years (youth), and 15–19 years ( adolescents).

Clinical variables included the length of stay (LOS) in acute care and special care days. LOS in acute care was defined as the number of days between admission and discharge. Special care days were defined as the cumulative number of days spent in all intensive care units.

Discharge disposition from acute care included death in acute care, discharge home (home, home with support services), and discharge to a non-home setting (inpatient rehabilitation, complex continuing care (CCC), long term care (LTC), and transferred to another inpatient setting).

### Analyses

Brain tumour episodes of care were used to determine the number and rate of healthcare utilization between fiscal years 2003/04 and 2009/10. The rationale for looking at episodes of care rather than hospitalizations only when assessing the burden of brain tumour on healthcare services is because a patient may not have a brain tumour diagnosis when admitted to the ED. By linking the DAD to the NACRS via a scrambled health card number, it is ensured that the population of interest is captured and that each episode was only captured once. This method of analysis has been shown to provide a more accurate description of the utilization of healthcare services [17]. Direct age- and sex-specific rates were generated by dividing the total number of brain tumour episodes by the population counts for the specific age group and sex to generate the number of brain tumour episodes of care for every 100,000 children and youth in Ontario, Canada.

Patient level analysis was used to examine the characteristics of patients with an ICD-10 brain tumour diagnostic code. This analysis captured only the patients’ initial brain tumour hospitalization between fiscal years 2003/04 and 2009/10, as a readmissions profile may differ from the initial admission. This is accomplished by using a look-back window of at least 1 year to ensure that the patients included were the initial hospitalization record between fiscal years 2003/04 and 2009/10. Because it was not possible to determine whether records identified in fiscal year 2003/04 were the initial hospitalization for a brain tumour (due to the lack of data to look back at least 1 year), this fiscal year of study was eliminated from the patient level analysis. This ensured that patients identified between fiscal years 2004/05 and 2009/10 were initial hospitalizations during this study period. Descriptive analyses were conducted and chi-squared tests and t-tests were used to compare patient characteristics and discharge destinations of patients with malignant and benign brain tumours and between patients with malignant and unspecified brain tumours.

### Ethics

Research ethics approval was obtained from the Toronto Rehabilitation Institute, University Health Network. Informed consent from patients is not possible as the data sources for this study are de-identified healthcare administrative databases.

## Results

### Rate of brain tumour episodes of care by age, sex, and fiscal year

**Fig. 1:**
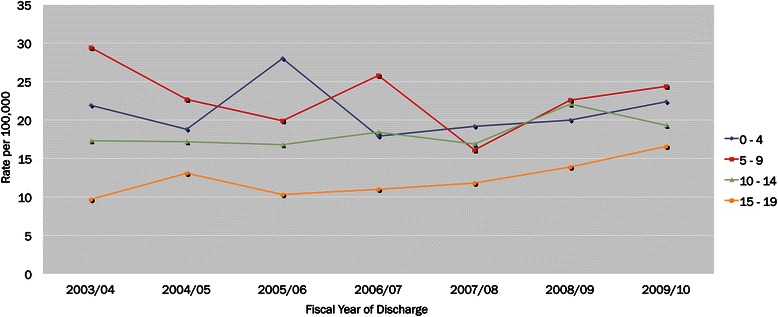
Rate of brain tumour episodes of care by fiscal year of discharge and age groups, Ontario, Canada, 2003/04–2009/10

Between fiscal years 2003/04 and 2009/10, there were 4022 brain tumour episodes of care (18.4 per 100,000 children and youth aged 19 years and under). The rate of brain tumour episodes of care was higher among males (20.7 per 100,000) compared to females (16.0 per 100,000). By age groups, the highest episodes of care were among children (23.1 per 100,000), followed by infants (21.2 per 100,000), youth (18.3 per 100,000), and adolescents (12.4 per 100,000). During this 7-year period, the rate of brain tumour episodes of care remained relatively steady, however, the rate among males fluctuated while the rate among females decreased from fiscal years 2003/04 to 2005/06 and increased from 2006/07 to 2009/10 (Fig. 1 and Additional file 1).

**Fig. 2:**
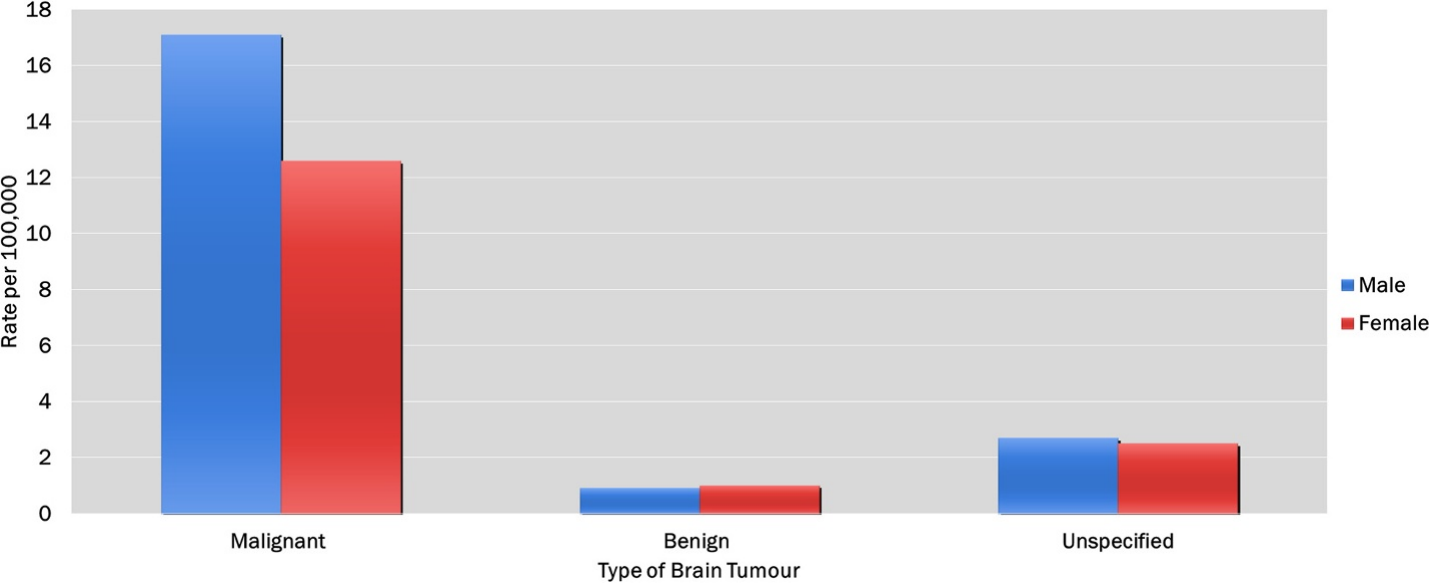
Rate of brain tumour episoes of care by type of brain tumour and sex, Ontario, Canada, 2003/04–2009/10

The rate of malignant brain tumour episodes of care during the study period was 14.9 per 100,000 (*n* = 3253) and was higher among males (17.1 vs. 12.6 per 100,000). By age groups, the highest rates were among children (19.8 per 100,000), followed by infants (17.2 per 100,000), youth (15.2 per 100,000), and adolescents (8.5 per 100,000). The rates of malignant brain tumour episodes of care decreased from fiscal years 2003/04 to 2007/08, after which it increased to rates seen in 2003/04 (Fig. 2 and Additional file 1).

Overall, there was a relatively low rate of benign brain tumour episodes of care (1.0 per 100,000; *n* = 211). The rate of benign brain tumours was slightly higher among females than males (1.0 vs. 0.9 per 100,000). The highest rates were among infants (1.4 per 100,000), followed by adolescents (1.3 per 100,000), and children and youth (0.6 per 100,000). The rates of benign brain tumour episodes of care increased between fiscal years 2003/04 and 2006/07, after which it decreased slightly (Fig. 2 and Additional file 1).

The rate of unspecified brain tumour episodes of care during the study period was 2.6 per 100,000 (*n* = 558) and was higher among males than females (2.7 vs. 2.4 per 100,000). By age groups, the rates were relatively similar - 2.5 per 100,000 among youth and adolescents, 2.6 per 100,000 among infants, and 2.7 per 100,000 among children. The rates of unspecified brain tumour episodes of care fluctuated during this study period (Fig. 2 and Additional file 1).

### Patient hospitalization characteristics

**Fig. 3:**
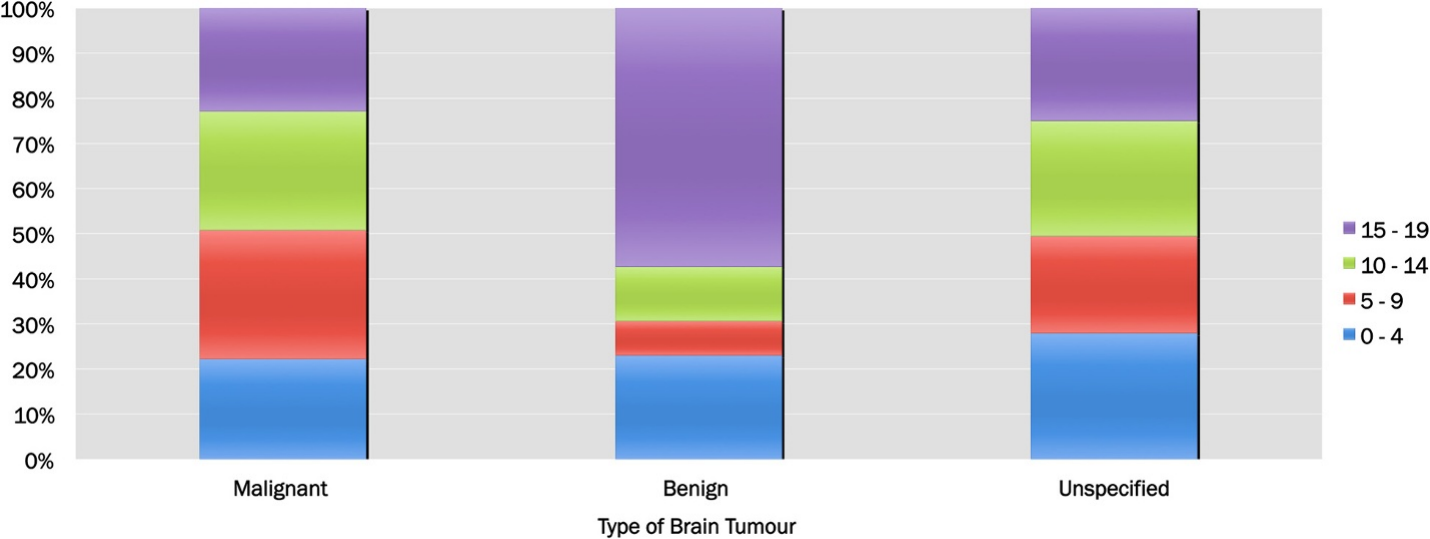
Distribution of age groups by type of brain tumour, Ontario, Canada, 2004/05–2009/10

Patient level analyses identified 745 children and youth with a brain tumour diagnostic code between fiscal year 2004/05 and 2009/10; 65.8 % had malignant brain tumours, 11.7 % had benign brain tumours, and 22.6 % had unspecified brain tumours. Overall and among those with malignant and unspecified brain tumours, the majority were males (55.0 % and 59.6 % respectively); however, the sex distribution of patients with benign brain tumours was approximately equal (Fig. 3 and Table 1).

**Table 1:** Demographic and clinical characteristics and discharge destinations of children and youth with brain tumours in acute care by type of brain tumour diagnosis, Ontario, Canada, 2004/05 - 2009/10

Characteristics	Overall		Malignant Brain Tumour		Benign Brain Tumour		Unspecified Brain Tumour	
	N	Col%	N	Col%	N	Col%	N	Col%
Overall	745	100	490	65.8	87	11.7	168	22.6
Age Groups								
0–4	174	23.4	109	22.2	18	20.7	47	28.0
5–9	182	24.4	140	28.6	6	6.9	36	21.4
10–14	190	25.5	129	26.3	18	20.7	43	25.6
15–19	199	26.7	112	22.9	45	51.7	42	25.0
Sex								
Males	410	55.0	267	54.5	43	49.4	100	59.5
Females	335	45.0	223	45.5	44	50.6	68	40.5
Length of Stay (Days)								
Average LOS (Mean, SD)	14.0	22.4	16.7	25.3	10.0	13.4	8.0	14.3
1–2	108	14.5	48	9.8	14	16.1	46	27.4
3–5	195	26.2	103	21.0	29	33.3	63	37.5
6–11	217	29.1	153	31.2	25	28.7	39	23.2
12+	225	30.2	186	38.0	19	21.8	20	11.9
Special Care Days								
Average Number of Special Care Days (Mean, SD)	4.7	12.8	4.2	11.6	3.3	5.0	9.1	21.6
None	377	50.6	218	44.5	38	43.7	121	72.0
1–2	247	33.2	182	37.1	32	36.8	33	19.6
3–5	69	9.3	52	10.6	9	10.3	8	4.8
6+	52	7.0	38	7.8	8	9.2	6	3.6
Discharge Disposition								
Home	619	83.1	418	85.3	81	92.1	120	71.4
Non-Home Setting	102	13.4	NR	.	6	6.9	NR	.
Death	24	3.2	NR	.	0	0	<5	.

*NR* not reportable due to small cell sizes

The average LOS in acute care was 14.0 days (SD = 22.4 days). Patients with malignant brain tumour had a significantly longer average LOS in acute care compared to patients with benign (*p* < .05) and unspecified brain tumours (*p* < .01). Slightly more than half of the patients with malignant and benign brain tumours and 28 % of patients with unspecified brain tumours had at least one special care day. Among those with special care days, 34 % of hospital days were spent in the intensive care units. Patients with unspecified brain tumours had a significantly longer stay in intensive care units compared to those with malignant brain tumours (*p* < .05; Table 2).

**Table 2:** Chi-squared test and t-test of select variables comparing malignant and benign brain tumours and malignant and unspecified brain tumours

Variable	MalignantMean (SD) or N (%)	Benign Mean (SD) or N (%)	*p*-value	MalignantMean (SD) or N (%)	UnspecifiedMean (SD) or N (%)	*p*-value
Average LOS	16.7 (25.3)	10.0 (13.4)	<.05	16.7 (25.3)	8.0 (14.3)	<.01
Special Care Days (1+)	272 (55.5)	49 (56.3)	0.9814	272 (55.5)	55 (32.7)	<.0001
Average Number of SCD	4.3 (11.6)	3.3 (5.0)	0.5939	4.2 (11.6)	9.1 (21.6)	<.01
Discharge Home	418 (85.3)	81 (92.1)	0.3367	418 (83.5)	120 (71.4)	<.0001

### Discharge destinations

Overall, 83.1 % were discharged home and 13.4 % to non-home settings; 3.2 % died in acute care. Chi-squared test showed that a significantly lower proportion of patients with unspecified brain tumours were discharged home (71.4 %; *p* < .0001) compared to patients with malignant brain tumours (83.5 %; Tables 1 and 2).

## Discussion

This is the first population-based paper, to the best of our knowledge, to capture metastatic, benign, and unspecified brain tumours in hospitalized children and youth and to provide detailed information on their healthcare utilization, including the number, trends, patient characteristics, and discharge destinations in Ontario, Canada. The number and rates of benign and unspecified brain tumour episodes of care identified in this study, despite being relatively low, provide evidence that current estimates of brain tumour episodes of care are underestimates, as they are limited to primary brain tumours. Therefore, it is expected that future estimates of the number and rates of brain tumours, when including metastatic, benign, and unspecified brain tumours, will be higher than currently reported in the literature. For example, in Australia in 2009, the rate of brain cancer (coded as ‘malignant neoplasm of the brain’) was less than five per 100,000, for both male and female under the age of 19 [18]. In the United States in 2000, the age-adjusted rate of patients aged 19 years and under with malignant brain tumours was 3.33 per 100,000 [19]. Data from this study showed that the combined brain tumour episode of care for primary, metastatic, benign, and unspecified brain tumour was 18.4 per 100,000 children and youth in Ontario. This is further supported by the findings that showed 12.1 % and 10.3 % of all pediatric brain tumours are classified as benign and uncertain, respectively [8]. A study in the United States that used additional information other than diagnostic coding to determine the type of brain tumour showed that approximately 22 % of all cases were non-malignant [8]. Further, it has been estimated that in 2002, the worldwide number of benign brain or central nervous system tumours was almost 200,000 cases, with crude estimated incidence rates of 4.9 per 100,000 [10]. Therefore, these findings, along with data presented in this paper, emphasize the need to include metastatic, benign, and unspecified brain tumours in surveillance and research. Metastatic brain cancer occurs in 20 to 40 % of all patients with cancer [20] and, even though it is not as common among children and youth [1], identifying metastatic brain tumours and their healthcare use is particularly important among survivors [21].

This paper also suggests the importance of accessing data sources from adult facilities when examining the children and youth population. In the cohort of benign brain tumours identified in this paper, 52 % of patients with benign brain tumours were adolescents aged 15–19 years of age. This is a population that is often missed either due to the transition from pediatric to adult healthcare facilities or because these adolescents are admitted to an adult facility [22]. Therefore, accessing data sources from adult facilities is important for research on children and youth to ensure that adolescents are not missed in surveillance or research.

Data from this study also showed that the overall rate of malignant brain tumour episode of care was approximately 14.9 times higher than that of benign brain tumours and 5.7 times higher than that of unspecified brain tumours. However, despite the higher rates of malignant brain tumour episodes of care, patients with benign and unspecified brain tumours also use acute care services that are currently not taken into account in resource allocation and healthcare planning. Compared to patients with malignant brain tumours, the proportion of patients with benign tumours that had at least one special care day, overall and by age group, was equally high (56 %), with similar average number of days spent in intensive care units. Of particular importance is that even though a significantly higher proportion of patients with malignant brain tumours had at least one special care day compared to those with unspecified brain tumours (56 % vs. 33 %), the average number of days spent in intensive care units was significantly higher in the unspecified tumour population, overall and in particular, among infants (32 days vs. 7 days). This finding highlights the fact that, as previously shown in the literature [8] there are patients that enter the acute care setting with an unknown type of brain tumour that, as expected, requires more intensive healthcare resources. It has been reported that in Canada, approximately 11 % of all urgent care centre visits in the emergency department were among patients aged 18 years and under [4]. Again, this proportion only takes into account primary brain tumours and only in the emergency department setting. As such, it is plausible that when taking into account metastatic, benign, and unspecified brain tumours and in the acute care setting, the number and proportion of patients that utilize urgent care centres in the emergency department and intensive care units may be much higher. In-depth research on children and youth with all types of brain tumours, by type of brain tumour, age group, and sex is needed to elucidate differences and similarities observed in this study. This has implications for the planning of targeted healthcare services for patients with primary, metastatic, benign, and unspecified brain tumours and in particular, the services that they require in the long-term.

Limitations associated with the use of administrative data must be recognized. First, while the patient level analysis included only the initial brain tumour related hospitalization between fiscal years 2004/05 and 2009/10 (vs. re-hospitalizations), they were determined with a look-back window of at least 1 year. As such, individuals identified in this study with an initial hospitalization record for brain tumour between fiscal years 2004/05 and 2009/10 may have been previously admitted to the hospital as recently as in fiscal year 2002/03. However, due to the lack of data available, it was not possible to determine the extent to which patients included in this study were previously admitted prior to fiscal year 2002/03. A longer look-back window may be more appropriate to detect changes in low-grade tumours and for various research questions. For example, it was found that a shorter look-back period of approximately 1 year is most appropriate for modeling mortality post-acute care discharge, however, longer look-back periods are optimal for readmission outcomes [23]. As such, additional research that follows these patients, particularly those with unspecified brain tumours, across the healthcare setting should be conducted. This includes readmissions to determine changes in diagnosis and healthcare use over time. Future studies with additional fiscal years of data will also be able to determine whether low-grade tumours have de-differentiated into higher-grade phenotypes by identifying the presence of this cohort of patients in subsequent fiscal years of data. Second, information on discharge destinations in this study were based on coded data in the DAD rather than actual linkage of records across the continuum of care and thus, misclassification bias is possible. Third, identification of brain tumours in this study used on ICD-10 codes rather than the WHO classification. While this limits our ability to extract detailed information on the brain tumour (e.g., histology, grade), a reabstraction study of the DAD indicated good agreement for non-clinical variables and moderate to substantial agreement for diagnoses [15]. As such, this paper is able to identify, with moderate to substantial accuracy, the presence of a brain tumour in the acute care setting, thus providing valuable population based information on the health service use and epidemiology of children and youth with brain tumours in Ontario, Canada. Fourth, information on first presentation and diagnosis are unavailable, as only the date of admission and discharge/transfer/deaths are recorded in the DAD and NACRS. Finally, this paper broadly classified patients with primary and metastatic brain tumour in the malignant brain tumour category due small cell sizes across age groups, sex, and fiscal years. It is acknowledged that these patients are treated differently and may have a different trajectory across the healthcare system. Future research, particularly on their health service use and outcomes across the healthcare setting, should stratify by primary and metastatic brain tumour.

Nonetheless, a strength of this study is that the data sources used for this study are population based; as such, this study captured all children and youth aged 19 years and under with a brain tumour ICD-10 diagnostic code in Ontario, Canada in the NACRS and the DAD. The use of the NACRS and the DAD also provided an opportunity identify brain tumour episodes of care rather than hospitalizations only, which has been shown to provide a more accurate description of the utilization of services, an objective of this study. It also ensured that each episode of care is captured only once (i.e., this ensured that no double counting occurred). Finally, case definition for brain tumour used in this study also included ICD-10 codes that indicated metastatic, benign, and unspecified brain tumours, which have neither been explored nor identified among children and youth in Ontario, Canada. Continued efforts to identify and include all patients with primary, metastatic, benign, and unspecified brain tumours are encourage. This may be accomplished by incorporating additional data sources, such as the Ontario Cancer Registry [24] that allow for the capturing of all patients with a primary brain tumour that may be missed if they do not visit the emergency department or seek acute care in Ontario. As discussed earlier, accessing data sources from adult healthcare facilities provide an opportunity to capture adolescents that may be admitted to an adult facility rather than a pediatric setting. Further, analyses stratified by primary and metastatic brain tumours should be conducted in order to identify differences in healthcare utilization and outcome of these populations. Finally, linkage of patients identified in the NACRS and DAD data to additional post-acute services data sources, such as homecare and physician services, can provide information on the trajectory of these patients across the healthcare continuum, including follow up services that are used post-acute care discharge.

## Conclusion

This is the first paper, to the best of our knowledge, to capture metastatic and non-malignant brain tumours in children and youth at a population based level. This allowed for a more accurate profile of health service utilization among children and youth with brain tumours. Further, this paper also identified additional in-depth information on the healthcare utilization of this hospitalized population, such as length of stay, special care days, and discharge destinations from acute care, which are currently absent even in the statistics on primary brain tumour in Canada. We acknowledge that primary and metastatic brain tumours are treated differently as well as malignant and non-malignant brain tumours. However, for the purpose of resource allocation and planning of healthcare services for all patients with brain tumours, the inclusion of metastatic and non-malignant brain tumours in surveillance and research is crucial. In particular, post-hospitalization services, including inpatient rehabilitation, may play an important role in the long-term recovery and functioning of children and youth with brain tumours, regardless of the type of brain tumour. Therefore, inclusion of metastatic and non-malignant brain tumours in the surveillance and research of brain tumours across the healthcare continuum can contribute to understanding the access and use of these services and can address barriers to, improve, and prepare healthcare services for this population.

## Additional file

Additional file 1: Table S1. Brain tumour episodes of care per 100,000 children and youth aged 19 years and under in Ontario between fiscal years 2003/04 and 2009/10 by age, fiscal year of discharge, and sex. Table S2 Brain tumour episodes of care per 100,000 children and youth aged 19 years and under in Ontario between fiscal years 2003/04 and 2009/10 by type of brain tumour, age, fiscal year of discharge, and sex. (DOCX 127 kb)

## Abbreviations

CIHI: Canadian Institute for Health Information
CCC: Complex continuing care
DAD: Discharge Abstract Database
ED: Emergency department
ICD-10: International Classification of Diseases version 10
LOS: Length of stay
LTC: Long-term care
NACRS: National Ambulatory Care Reporting System
