# Supplementary material for: A population based perspective on children and youth with brain tumours
Source: BMC Cancer. 2015 Dec 23;15:1007. doi: 10.1186/s12885-015-2016-0 (PMC4690355; doi:10.1186/s12885-015-2016-0)
Supplement: Additional file 1: Table S1. — Brain tumour episodes of care per 100,000 children and youth aged 19 years and under in Ontario between fiscal years 2003/04 and 2009/10 by age, fiscal year of discharge, and sex. Table S2 Brain tumour episodes of care per 100,000 children and youth aged 19 years and under in Ontario between fiscal years 2003/04 and 2009/10 by type of brain tumour, age, fiscal year of discharge, and sex. (DOCX 127 kb) [file 12885_2015_2016_MOESM1_ESM.docx]

**Table 1.** Brain tumour episodes of care per 100,000 children and youth aged 19 years and under in Ontario between fiscal years 2003/04 and 2009/10 by age, fiscal year of discharge, and sex.

|  | **Overall** | | | **0 – 4** | | | **5 – 9** | | | **10 – 14** | | | **15 – 19** | | |
| --- | --- | --- | --- | --- | --- | --- | --- | --- | --- | --- | --- | --- | --- | --- | --- |
|  | **Overall**  n  rate | **Male**  n  Rate | **Female**  n  rate | **Overall**  n  rate | **Male**  n  rate | **Female**  n  rate | **Overall**  n  rate | **Male**  n  rate | **Female**  n  rate | **Overall**  n  rate | **Male**  n  rate | **Female**  n  rate | **Overall**  n  rate | **Male**  n  rate | **Female**  n  rate |
| 2003/04 | 605  19.3 | 364  22.7 | 241  15.8 | 150  21.9 | 102  29.2 | 48  14.4 | 230  29.4 | 108  27.1 | 122  31.8 | 145  17.3 | 104  24.3 | 41  10.0 | 80  9.7 | 50  11.7 | 30  7.4 |
| 2004/05 | 557  17.8 | 331  20.7 | 226  14.8 | 128  18.8 | 81  23.2 | 47  14.1 | 174  22.7 | 83  21.2 | 91  24.2 | 145  17.2 | 105  24.4 | 40  9.7 | 110  13.1 | 62  14.3 | 48  11.7 |
| 2005/06 | 569  18.2 | 360  22.5 | 209  13.7 | 191  28.0 | 110  31.4 | 81  24.4 | 149  19.9 | 84  22.0 | 65  17.7 | 141  16.8 | 105  24.5 | 36  8.8 | 88  10.3 | 61  13.8 | 27  6.5 |
| 2006/07 | 562  18.0 | 334  20.8 | 228  14.9 | 124  17.9 | 50  14.0 | 74  21.9 | 189  25.8 | 122  32.6 | 67  18.8 | 153  18.4 | 92  21.7 | 61  15.0 | 96  11.0 | 70  15.6 | 26  6.1 |
| 2007/08 | 492  15.8 | 257  16.1 | 235  15.5 | 134  19.2 | 55  15.4 | 79  23.4 | 116  16.1 | 69  18.6 | 47  13.4 | 138  16.9 | 71  17.0 | 67  16.8 | 104  11.8 | 62  13.8 | 42  9.8 |
| 2008/09 | 602  19.4 | 345  21.7 | 257  17.0 | 140  20.0 | 90  25.0 | 50  14.7 | 162  22.6 | 103  28.0 | 59  16.9 | 177  22.1 | 81  19.8 | 96  24.5 | 123  13.9 | 71  15.7 | 52  12.0 |
| 2009/10 | 635  20.4 | 322  20.2 | 313  20.6 | 154  22.4 | 90  25.5 | 64  19.1 | 177  24.4 | 87  23.5 | 90  25.4 | 158  19.3 | 69  16.5 | 89  22.2 | 146  16.6 | 76  16.9 | 70  16.4 |
| 2003/04 – 2009/10 | 4022  18.4 | 2313  20.7 | 1709  16.0 | 1021  21.2 | 578  23.3 | 443  18.9 | 1197  23.1 | 656  24.7 | 541  21.3 | 1057  18.3 | 627  21.2 | 430  15.2 | 747  12.4 | 452  14.6 | 295  10.0 |

**Table 2.** Brain tumour episodes of care per 100,000 children and youth aged 19 years and under in Ontario between fiscal years 2003/04 and 2009/10 by type of brain tumour, age, fiscal year of discharge, and sex.

|  | **Overall** | | | **0 – 4** | | | **5 – 9** | | | **10 – 14** | | | **15 – 19** | | |
| --- | --- | --- | --- | --- | --- | --- | --- | --- | --- | --- | --- | --- | --- | --- | --- |
|  | **Malignant**  n  rate | **Benign**  n  rate | **Unspecified**  n  rate | **Malignant**  n  rate | **Benign**  n  rate | **Unspecified**  n  rate | **Malignant**  n  Rate | **Benign**  n  rate | **Unspecified**  n  rate | **Malignant**  n  rate | **Benign**  n  rate | **Unspecified**  n  rate | **Malignant**  n  rate | **Benign**  n  rate | **Unspecified**  n  rate |
| 2003/04 | 506  16.2 | 22  0.7 | 77  2.5 | 130  19.0 | NR | 14  2.0 | 202  25.8 | NR | 22  2.8 | 124  14.8 | NR | 15  1.8 | 50  6.0 | NR | 26  3.1 |
| 2004/05 | 452  14.4 | 25  0.8 | 80  2.6 | 103  15.1 | NR | 22  3.2 | 147  19.2 | NR | 21  2.7 | 135  16.0 | NR | 8  1.0 | 67  8.0 | NR | 29  3.4 |
| 2005/06 | 458  14.6 | 27  0.9 | 84  2.7 | 156  22.9 | NR | 23  3.4 | 133  17.7 | NR | 14  1.9 | 112  13.3 | NR | 26  3.1 | 57  6.6 | NR | 21  2.4 |
| 2006/07 | 458  14.6 | 39  1.2 | 65  2.1 | 86  12.4 | NR | 22  3.2 | 173  23.7 | NR | 14  1.9 | 128  15.4 | NR | 17  2.0 | 71  8.1 | NR | 12  1.4 |
| 2007/08 | 371  11.9 | 37  1.2 | 84  2.7 | 98  14.1 | NR | 20  2.9 | 89  12.3 | NR | 22  3.0 | 107  13.1 | NR | 27  3.3 | 77  8.7 | NR | 15  1.7 |
| 2008/09 | 496  16.0 | 29  0.9 | 77  2.5 | 123  17.6 | NR | 12  1.7 | 138  19.2 | NR | 15  2.1 | 142  17.7 | NR | 30  3.7 | 93  10.5 | NR | 20  2.3 |
| 2009/10 | 512  16.5 | 32  1.0 | 91  2.9 | 132  19.2 | NR | 12  1.7 | 147  20.3 | NR | 30  4.1 | 131  16.0 | NR | 19  2.3 | 102  11.6 | NR | 30  3.4 |
| 2003/04 – 2009/10 | 3253  14.9 | 211  1.0 | 558  2.6 | 828  17.2 | 68  1.4 | 125  2.6 | 1029  19.8 | 30  0.6 | 138  2.7 | 879  15.2 | 36  0.6 | 142  2.5 | 517  8.5 | 77  1.3 | 153  2.5 |
| **Male** | | | | | | | | | | | | | | | |
| 2003/04 | 306  19.1 | 17  1.1 | 41  2.6 | 91  26.1 | NR | 7  2.0 | 93  23.3 | NR | 10  2.5 | 90  21.0 | NR | 8  1.9 | 32  7.5 | NR | 16  3.8 |
| 2004/05 | 278  17.3 | 14  0.9 | 39  2.4 | 66  18.9 | NR | 12  3.4 | 71  18.2 | NR | 9  2.3 | 99  23.0 | NR | NR  - | 42  9.7 | NR | NR  - |
| 2005/06 | 291  18.2 | 10  0.6 | 59  3.7 | 91  26.0 | NR | 14  4.0 | 73  19.1 | NR | NR  - | 84  19.6 | NR | NR  - | 43  9.8 | NR | 16  3.6 |
| 2006/07 | 286  17.9 | 13  0.8 | 35  2.2 | 37  10.4 | NR | 10  2.8 | 117  31.2 | NR | NR  - | 77  18.2 | NR | 11  2.6 | 55  12.3 | NR | NR  - |
| 2007/08 | 192  12.0 | 16  1.0 | 49  3.1 | 40  11.2 | NR | 12  3.4 | 52  14.0 | NR | 12  3.2 | 54  12.9 | NR | 15  3.6 | 46  10.2 | NR | 10  2.2 |
| 2008/09 | 297  18.7 | 17  1.1 | 31  1.9 | 82  22.8 | NR | 5  1.4 | 88  23.9 | NR | 7  1.9 | 68  16.6 | NR | 11  2.7 | 59  13.0 | NR | 8  1.8 |
| 2009/10 | 265  16.7 | 13  0.8 | 44  2.8 | 81  23.0 | NR | 6  1.7 | 70  18.9 | NR | 17  4.6 | 58  13.9 | NR | 6  1.4 | 56  12.4 | NR | 15  3.3 |
| 2003/04 – 2009/10 | 1915  17.1 | 100  0.9 | 298  2.7 | 488  19.7 | 24  1.0 | 66  2.7 | 564  21.2 | 22  0.8 | 70  2.6 | 530  17.9 | 22  0.7 | 75  2.5 | 333  10.7 | 32  1.0 | 87  2.8 |
| **Female** | | | | | | | | | | | | | | | |
| 2003/04 | 200  13.1 | 5  0.3 | 7  2.1 | 39  11.7 | NR | 7  2.1 | 109  18.4 | NR | 12  3.1 | 34  8.3 | NR | 7  1.7 | 18  4.5 | NR | 10  2.5 |
| 2004/05 | 174  11.4 | 11  0.7 | 10  3.0 | 37  11.1 | NR | 10  3.0 | 76  20.2 | NR | 12  3.2 | 36  8.7 | NR | <5  - | 25  6.1 | NR | NR  - |
| 2005/06 | 167  10.9 | 17  1.1 | 9  2.7 | 65  19.6 | NR | 9  2.7 | 60  16.4 | NR | <5  - | 28  6.8 | NR | NR  - | 14  3.4 | NR | 5  1.2 |
| 2006/07 | 172  11.3 | 26  1.7 | 12  3.6 | 49  14.5 | NR | 12  3.6 | 56  15.7 | NR | NR  - | 51  12.5 | NR | 6  1.5 | 16  3.8 | NR | <5  - |
| 2007/08 | 179  11.8 | 21  1.4 | 8  2.4 | 58  17.2 | NR | 8  2.4 | 37  10.5 | NR | 10  2.8 | 53  13.3 | NR | 12  3.0 | 31  7.2 | NR | 5  1.2 |
| 2008/09 | 199  13.1 | 12  0.8 | 7  2.1 | 41  12.1 | NR | 7  2.1 | 50  14.3 | NR | 8  2.3 | 74  18.9 | NR | 19  4.8 | 34  7.8 | NR | 12  2.8 |
| 2009/10 | 247  16.3 | 19  1.3 | 6  1.8 | 51  15.3 | NR | 6  1.8 | 77  21.8 | NR | 13  2.7 | 73  18.2 | NR | 13  3.2 | 46  10.8 | NR | 15  3.5 |
| 2003/04 – 2009/10 | 1338  12.6 | 111  1.0 | 59  2.5 | 340  14.5 | 44  1.9 | 59  2.5 | 465  18.3 | 8  0.3 | 68  2.7 | 349  12.3 | 14  0.5 | 67  2.4 | 184  6.2 | 45  1.5 | 66  2.2 |

Note: NR = not reportable due to small cell sizes
